# Metabolome, microbiome, and gene expression alterations in the colon of newborn piglets with intrauterine growth restriction

**DOI:** 10.3389/fmicb.2022.989060

**Published:** 2022-09-14

**Authors:** Wu Tang, Wanghong Zhang, Md. Abul Kalam Azad, Cui Ma, Qian Zhu, Xiangfeng Kong

**Affiliations:** ^1^Hunan Provincial Key Laboratory of Animal Nutritional Physiology and Metabolic Process, Key Laboratory of Agro-Ecological Processes in Subtropical Region, National Engineering Laboratory for Pollution Control and Waste Utilization in Livestock and Poultry Production, Institute of Subtropical Agriculture, Chinese Academy of Sciences, Changsha, Hunan, China; ^2^College of Advanced Agricultural Sciences, University of Chinese Academy of Sciences, Beijing, China

**Keywords:** colonic microbiota, intrauterine growth restriction, metabolism, piglets, short-chain fatty acids

## Abstract

Newborn animals with intrauterine growth restriction (IUGR) are characterized by impaired intestinal structure and function; however, their intestinal microbiota and metabolome profiles have not been fully identified. The present study investigated the differences in colonic microbiota, metabolomics, and barrier function-related gene expression profiles between the IUGR and normal birth weight (NBW) piglets at 7, 21, and 28 days of age. Forty-eight piglets (24 NBW and 24 IUGR) from 24 litters were assigned to assess the differences in colonic microbiota, metabolomics, and gene expression between IUGR and NBW piglets. Compared with the NBW piglets, IUGR piglets showed decreased Shannon index and increased Simpson index at 7 days of age and Chao1 index at 21 days of age (*p* < 0.05). The IUGR piglets had lower abundances of Firmicutes, *Subdoligranulum*, *Ruminococcaceae_UCG-002*, and *Ruminococcaceae_UCG-003* at 7 days of age, and Bacteroidetes, *Phascolarctobacterium*, and *Ruminococcaceae_UCG-005* at 21 days of age, when compared with the NBW piglets (*p* < 0.05). Metabolomics analysis showed significant changes in 147 metabolites mainly involved in organic acids and their derivatives in the colon. Six differential metabolic pathways were significantly enriched, including purine metabolism, amino sugar/nucleotide sugar metabolism, ubiquinone/other terpenoid-quinone biosynthesis, phenylalanine/tyrosine/tryptophan biosynthesis, phenylalanine metabolism, and histidine metabolism. Spearman’s correlation analysis further demonstrated significant correlations between colonic microbiota and metabolites. In addition, colonic isobutyrate at 7 days of age, isovalerate and total short-chain fatty acids (SCFAs) at 21 days of age, and acetate, propionate, butyrate, and total SCFAs levels at 28 days of age were lower and isovalerate was higher at 28 days of age in the IUGR piglets than in the NBW piglets (*p* < 0.05). Furthermore, the mRNA expression of *zonula occludens* (*ZO*)-1 at 7 days of age, *ZO*-1, *occludin*, and interleukin (*IL*)-4 at 21 days of age were down-regulated in the IUGR piglets, whereas tumor necrosis factor (*TNF*)-α and nuclear factor-kappa B (*NF-κB*) at 28 days of age were up-regulated, when compared with the NBW piglets (*p* < 0.05). The findings suggest that the IUGR pigs present abnormal microbiota and nutrient metabolism in the colon, which may further affect the intestine barrier function by regulating gene expressions.

## Introduction

Intrauterine growth restriction (IUGR) has been defined as the impaired growth and development of the mammalian embryo/fetus or its organs during pregnancy (D'Inca et al., 2010). There are several factors, including genetic factors, environmental stress, insufficient uterine capacity, and maternal malnutrition, which could induce the IUGR of piglets (Wu et al., 2004). The IUGR can lead to feeding intolerance, decreased fat absorption, and digestive disorders in the early life of pigs (Gilbert and Danielsen, 2003), resulting in higher perinatal mortality and morbidity (Wu et al., 2006). Newborn piglets with IUGR have been identified with impaired intestinal structure and functions, resulting from the developmental pattern changes in their intestinal structure, transcriptomic, and proteomic profiles (Dong et al., 2016). Therefore, IUGR has attracted increasing attention in animal production (Ferenc et al., 2017).

The intestine is not only an important organ for nutrient digestion, absorption, and metabolism but also the largest immune organ protecting against pathogens in animals. The mammalian intestine is the harbor of microbes and is associated with a broad range of functions within the host. For example, the intestinal microbiota participates in the fermentation of complex carbohydrates, production of nutrients and vitamins, protection against pathogens, maintenance of immune balance, and different nutrient metabolism of the host (Marchesi et al., 2016). Early colonization and development of piglets’ intestinal microbiota is a dynamic process characterized by rapid changes in microbial diversity, composition, and abundance (Matamoros et al., 2013). Several factors, such as delivery mode, diet, living environment, and diseases, can affect the early colonization and development of the intestinal microbiota of piglets (Nicholson et al., 2012). Emerging evidence confirmed that the colonization, succession process, and the balance of intestinal microbiota directly affect the intestinal barrier function (Kamada et al., 2013), metabolic reactions (Sonnenburg and Backhed, 2016), trophic effects (Sonnenburg and Backhed, 2016), and maturation of immune responses of the host (Littman and Pamer, 2011). In addition, intestinal microbiota can ferment undigested dietary components and endogenous compounds and produce short-chain fatty acids (SCFAs; Rooks and Garrett, 2016). Therefore, the gut microbiota balance and normal metabolic status are closely related to the host’s health (Valdes et al., 2018).

Previous studies showed that the intestinal microbial community diversity of IUGR piglets is lower than the normal birth weight (NBW) piglets, which is easier to affect by pathogens (Jiang et al., 2019). In addition, newborn piglets with IUGR had lower microbial diversity and different taxonomic abundances in the small intestine (Zhang et al., 2019). Li et al. (2018) found that the low-birth-weight piglets also had different fecal microbial community structures and metabolome profiles. These findings suggested that the alteration of the intestinal microbiota is potentially associated with impaired growth and development of piglets. The colon is the main site of microbial fermentation in pigs (Sciascia et al., 2016), and numerous small molecular compounds are produced in this process, which affects the gene expressions related to the intestinal function of the host (Chassaing et al., 2017). Thus, we hypothesized that there might be differences in colonic microbiota and metabolites between the IUGR and NBW piglets, which may alter the intestinal barrier function-related gene expressions. Therefore, the present study was conducted to compare the differences in colonic microbiota, metabolites, and barrier function-related gene expression levels between IUGR and NBW piglets at 7, 21, and 28 days of age, using 16S rRNA gene sequencing and metabolomics technology to identify the biomarkers of intestinal microbiota and their metabolites.

## Materials and methods

### Experimental design and sample collection

Twenty-four pregnant sows with similar physical conditions with 3–5 parities were selected and herded in a pig farm located in Yongan town, Liuyang city, Hunan province, China. The sows were housed individually in gestation crates (2.2 × 0.6 m) from day 1 to day 105 of pregnancy and then housed in farrowing crates (2.2 × 1.8 m) until weaning. The sows were fed ~3 kg of food twice (at 8:00 and 17:00 h) daily during the experimental period, and the diet fluctuated with the sows’ physical condition. Experimental sows had free access to drink water at all times. A total of 48 (male:female, 1:1) Large White × Landrace newborn piglets (24 NBW, 1.68 ± 0.04 kg and 24 IUGR, 0.98 ± 0.02 kg body weight; BW) with one NBW piglet and one IUGR piglet from 24 litters were selected for this trial. Piglets with a heavier birth weight than the average birth weight per litter were identified as the NBW piglets, while those with a lower birth weight by 10% than the average birth weight of their origin were defined as the IUGR piglets (Bauer et al., 1998). Suckling piglets were kept in a warm thermal container and fed by sows freely. Piglets received commercial creep feed from 5 days of age. The piglets were weaned at 21 days of age and transferred to nursery facilities with *ad libitum* access to water and weaning diets at all times. No antibiotics were used during the experimental period. All male piglets were castrated.

**Figure 1:**
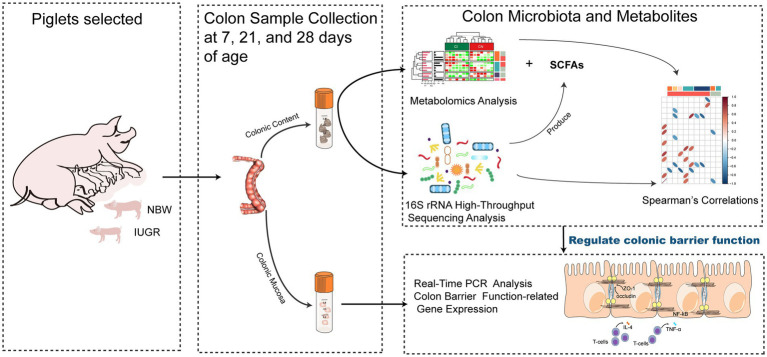
Schematic presentation of the overall experimental procedure on metabolome, microbiome, and gene expression alterations in colon of newborn piglets with intrauterine growth restriction.

At 7 (IUGR = 1.77 ± 0.08 kg and NBW = 2.79 ± 0.16 kg; BW), 21 (IUGR = 4.71 ± 0.41 kg and NBW = 6.41 ± 0.35 kg; BW), and 28 (IUGR = 5.06 ± 0.52 kg and NBW = 7.96 ± 0.34 kg; BW) days of age, 16 suckling piglets (eight pairs with one NBW piglet and one IUGR littermate) were weighed 2 h after the last suckling and then euthanized after anesthetization with sodium pentobarbital (40 mg/kg BW, Shanghai Haling Biological Technology Co., Ltd., Shanghai, China) for colonic contents and mucosa collection. The colonic contents (2 cm above the terminal colon) were collected and immediately stored at −80°C for analysis of the microbial composition, metabolome profiles, and SCFAs. The mucosa (~2 g) were sampled, quickly frozen into liquid nitrogen, and stored at −80°C for analysis of gene expressions. The overall experimental procedure is presented in Figure 1.

### DNA extraction, Illumina MiSeq sequencing, and bioinformatics analysis

Microbial DNA of the colonic contents was extracted using HiPure Stool DNA Kit (Magen, Guangzhou, China), following the manufacturer’s instructions. The final concentration and purity of the extracted DNA were determined using a NanoDrop 2000 UV–vis spectrophotometer (Thermo Fisher Scientific, Waltham, MA, Unites States), and DNA quality was checked by 1% agarose gel electrophoresis. The V3-V4 hypervariable regions of the 16S rRNA gene were amplified with primers 338F (5′-ACTCCTACGGGAGGCAGCAG-3′) and 806R (5′-GGACTACHVGGGTWTCTAAT-3′) using thermocycler polymerase chain reaction (PCR) system (GeneAmp 9700, ABI, Unites States). The PCR reactions were conducted as described previously (Zhang et al., 2019). The resulting PCR products were extracted using 2% agarose gel electrophoresis, then purified using an AxyPrep DNA Gel Extraction Kit (Axygen Biosciences, Union City, CA, Unites States) and quantified using QuantiFluor™-ST (Promega, Madison, WI, Unites States) following the manufacturer’s protocols. The equimolar purified amplicons were pooled, and paired-end (2 × 300) sequenced on an Illumina MiSeq platform (Illumina, San Diego, CA, Unites States), following the standard protocols by Majorbio Bio-Pharm Technology Co., Ltd. (Shanghai, China).

Raw fastq files were demultiplexed, quality-filtered by Trimmomatic, and merged by FLASH 1.2.11 (Magoc and Salzberg, 2011) with the following criteria: (i) the reads were truncated at any site receiving an average quality score < 20 and over a 50 bp sliding window; (ii) primers were exactly matched with allowing two nucleotides to mismatch, and reads containing ambiguous bases were removed; (iii) sequences of overlap >10 bp were merged according to their overlap sequence. The operational taxonomic units (OTUs) were clustered with a 97% similarity cutoff using UPARSE 7.1 and chimeric sequences were identified and removed using UCHIME (Edgar, 2013, 2016). The taxonomy of each 16S rRNA gene sequence was analyzed by RDP 11.5 Classifier algorithm against the SILVA 128 database using a confidence threshold of 70% (Wang et al., 2007). Raw sequences obtained in this study are deposited in the NCBI Sequence Read Archive database with accession number PRJNA836103.^1^ Alpha diversity analysis included Shannon, Simpson, Chao1 richness estimator, and abundance-based coverage estimator (ACE) metric. Beta diversity analysis was performed to investigate the structural variation of microbial communities among samples using the principal coordinate analysis (PCoA). Partial least squares-discriminant analysis (PLS-DA) was also used as a supervised model to reveal the microbiota variation between the two groups. The taxonomic composition was investigated at the phylum and genus levels. Microbial functions were predicted by the phylogenetic investigation of communities by reconstruction of unobserved states one (PICRUSt1). All analyses were performed on the free online platform of Majorbio I-Sanger Cloud Platform.^2^

### Metabolite extraction, LC–MS analysis, and data processing

Colon contents (25 mg per sample) were weighed into 2 ml Eppendorf tubes (Eppendorf, Hamburg, Germany) and mixed with 500 μl extract solution (methanol: acetonitrile: water = 2:2:1 (v/v), containing isotopically labeled internal standard mixture). Then, the solution was vortexed for 30 s, homogenized at 35 Hz for 4 min, and sonicated for 5 min in the ice-water bath. The above treatment was repeated three times. After incubation at −40°C using a low-temperature freezer (AUCMA, Qingdao, China) for 1 h, the mixture solution was centrifuged at 10,000 × *g* and 4°C for 15 min, and then the resulting supernatant was transferred to a fresh glass vial for analysis. The quality control (QC) sample was prepared by mixing an equal aliquot of the supernatants from all samples.

The LC–MS/MS analyses were performed using an UHPLC system (Vanquish, Thermo Fisher Scientific, MA, Unites States) with an UPLC BEH Amide column (2.1 × 100 mm, 1.7 μm) coupled with Q Exactive HFX mass spectrometer (Orbitrap MS, Thermo Fisher Scientific). The mobile phase consisted of 25 mmol/L ammonium acetate, 25 mmol/L ammonia hydroxide in water (pH = 9.75), and acetonitrile. The auto-sampler temperature was 4°C, and the injection volume was 3 μl. The Q Exactive HFX mass spectrometer was used for its ability to acquire MS/MS spectra on information-dependent acquisition (IDA) mode in the control of the acquisition software (Xcalibur, Thermo Fisher Scientific). The Electrospray ion (ESI) source conditions were set as follows: sheath gas flow rate as 30 Arb, Aux gas flow rate as 25 Arb, capillary temperature as 350°C, full MS resolution as 60,000, MS/MS resolution as 7,500, collision energy as 10/30/60 in NCE mode, and spray voltage as 3.6 kV (positive, POS) or −3.2 kV (negative, NEG), respectively.

The raw data were converted to mzXML format using ProteoWizard and processed with an in-house program, which was developed using R package and based on XCMS, for peak detection, extraction, alignment, and integration. Then an in-house secondary mass spectrometry (MS2) database (BiotreeDB V 2.1) was applied in metabolite annotation. The cutoff for annotation was set at 0.3. The SIMCA (V16.0.2, Sartorius Stedim Data Analytics AB, Umea, Sweden) was used for principal component analysis (PCA) and orthogonal partial least squares discriminant analysis (OPLS-DA). Seven-fold cross-validation was used to examine the quality of the model. Permutation tests were used to test the validity of the model. The first principal component of variable importance in the projection (VIP) and Student’s *t*-test were obtained to refine the analysis. If VIP > 1 and *p* < 0.05, the variable was defined as a significantly different metabolite (SDM) between the two groups. In addition, the Kyoto Encyclopedia of Genes and Genomes (KEGG, http://www.genome.jp/kegg/) and MetaboAnalyst^3^ databases were used for pathway enrichment analysis.

### Quantitation of short-chain fatty acids in colonic contents

The levels of colonic SCFAs, including acetate, propionate, isobutyrate, butyrate, isovalerate, and valerate, were detected by using Agilent 6890 gas chromatography (Agilent Technologies, Inc., Palo Alto, CA, Unites States), as described previously (Zhou et al., 2012). Briefly, colonic contents samples (~1 g) were homogenized and centrifuged in sealed tubes at 10,000 × *g* and 4°C for 10 min. A mixture of the supernatant fluid and 25% metaphosphoric acid solution (1:0.25 ml) was filtered through a 0.45-μm polysulfone microporous membrane filter and then analyzed.

### RNA extraction and barrier function-related gene expression analysis

Total RNA extraction and real-time PCR measurement were conducted as previously described (Duan et al., 2017). Briefly, the total RNA of the colonic samples was extracted using the TRIZOL reagent (Magen, Guangzhou, China). The purity and concentration of the extracted RNA were assessed using a NanoDrop ND-2000 spectrophotometer (Thermo Fisher Scientific). The total RNA was reverse-transcribed into cDNA using a PrimeScript RT reagent kit with gDNA Eraser (TaKaRa Biotech. Co., Ltd., Dalian, China). An RT-PCR assay was carried out using the SYBR^®^ Premix Ex Taq™ kit (TaKaRa Biotech. Co., Ltd.) on a 480 II system (Roche, Light Cycler^®^ 480 II, Basel, Switzerland) with the following conditions: initial denaturation at 95°C for 30 s, followed by 40 cycles of denaturation at 95°C for 5 s, annealing at 60°C for 30 s, and a final extension at 72°C for 30 s. The primers for barrier function-related gene expression and reference gene β-actin (listed in Supplementary Table 1) were designed using the Primer-BLAST. The relative expression level of each target gene was calculated using the 2^−∆∆Ct^ method (Schmittgen and Livak, 2008).

### Statistical analysis

Student’s *t*-test was used for the significance test of metabolites and gene expression data. Mann–Whitney *U* test was used for the microbiota data. Differences between the IUGR and NBW piglets were considered significant at *p* < 0.05. Spearman’s correlation coefficient was used to assess the relationships with different microbiota, SDMs, and BW between the IUGR and NBW Piglets. Data are presented as means ± SEM (standard error of the mean). The SPSS 22.0 (SPSS Inc., Chicago, IL, Unites States), R packages ggplot2 V3.3.5 and ComplexHeatmap V2.10.0,^4^ and GraphPad Prism V.6.0 (San Diego, CA, Unites States) were used for data analysis and graph preparation.

## Results

### Differences in colonic microbial community diversity between IUGR and NBW piglets

**Figure 2:**
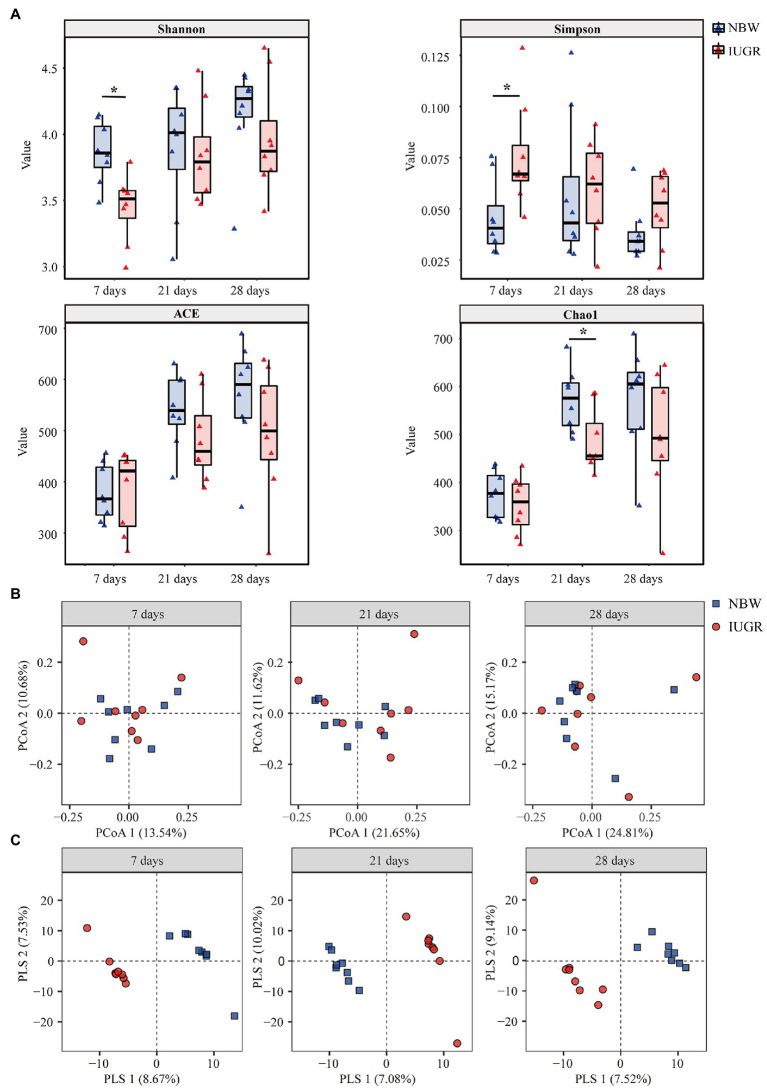
Differences in colonic microbial community diversity between the intrauterine growth restriction (IUGR) and normal birth weight (NBW) piglets at 7, 21, and 28 days of age. **(A)** The microbial Alpha diversity was measured using the Shannon, Simpson, ACE, and Chao indexes (*n* = 8). **(B)** Principal coordinate analysis (PCoA) on unweighted UniFrac distances (*n* = 8). **(C)** Partial least square discriminant analysis (PLS-DA) score plots of colonic microbiota (*n* = 8). The IUGR group and NBW group are shown along the first two PCoA/PLS axes. Each symbol represents the gut microbiota of a piglet, red represents the IUGR group, and blue represents the NBW group. ^*^p < 0.05.

The microbial community diversity differences between the IUGR and NBW piglets are presented in Figure 2. A total of 2,832,611 high-quality reads were generated by high-throughput sequencing of 48 samples. From the high-quality reads, 1095 OTUs were detected by clustering non-repetitive sequences based on 97% similarity, including 19 phyla, 32 classes, 51 orders, 93 families, 170 genera, and 216 species. Each sample contained with an average of 50,447 sequences and 47,047 OTUs. The rarefaction curves reached the peak, indicating a near-complete sampling of the colonic microbial community (Supplementary Figure 1).

Alpha diversity was measured using the Shannon, Simpson, ACE, and Chao1 indexes (Figure 2A). The IUGR piglets had a lower (*p* < 0.05) Shannon index and a higher Simpson index at 7 days of age compared with the NBW piglets. In addition, the IUGR piglets had a lower (*p* < 0.05) Chao1 index compared with the NBW piglets at 21 days of age.

The PCoA results showed that there was no obvious separation between the IUGR and NBW piglets at 7, 21, and 28 days of age (Figure 2B). The PLS-DA showed that the intergroup score plots were clearly separated and clustered into two groups at 7, 21, and 28 days of age, indicating that there were significant differences in the colonic microbial structure between the IUGR and NBW piglets (Figure 2C).

### Differences in colonic microbial composition between IUGR and NBW piglets

**Figure 3:**
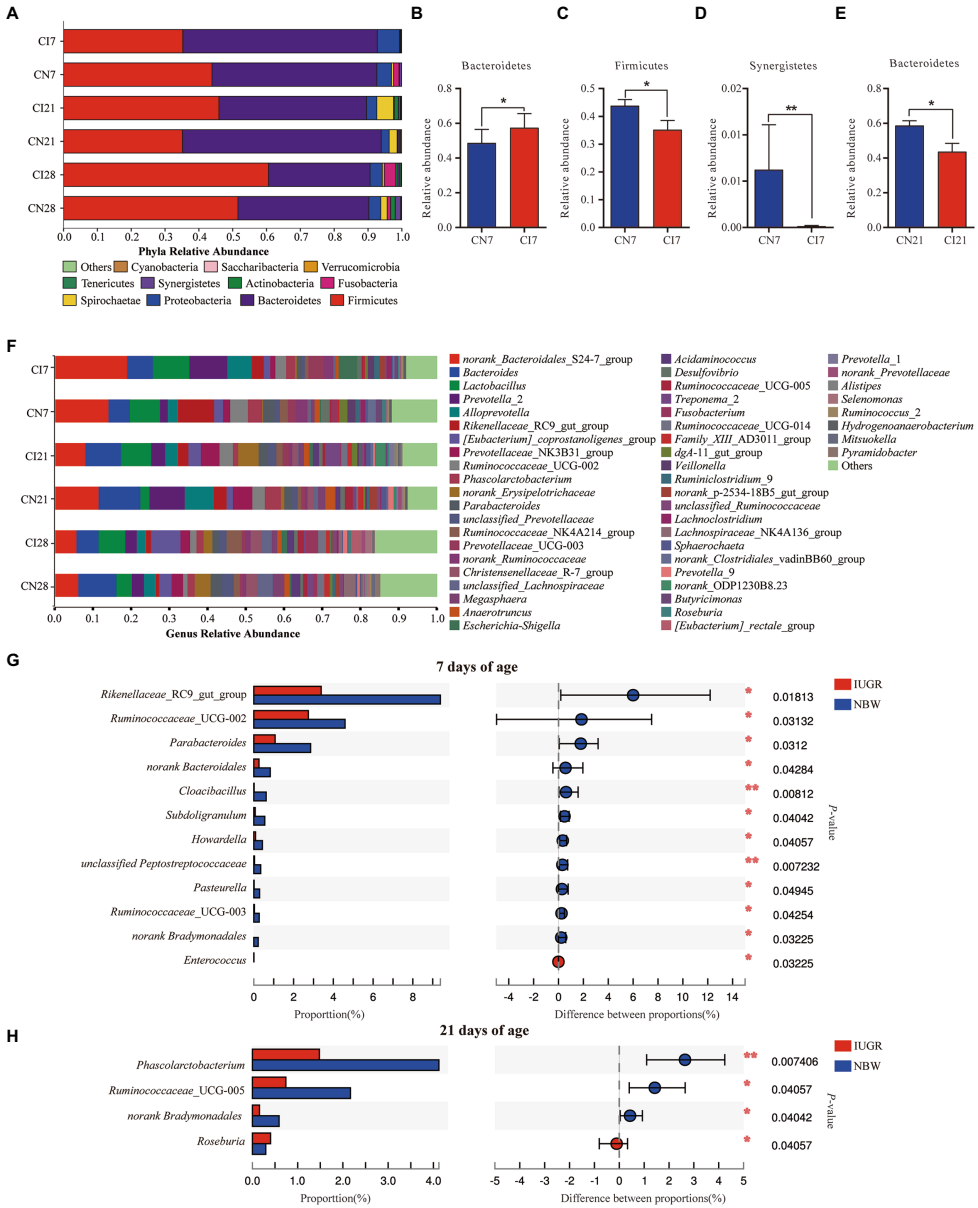
The Colonic microbial composition and taxonomic differences between the intrauterine growth restriction (IUGR) and normal birth weight (NBW) piglets at 7, 21, and 28 days of age. **(A)** The relative abundances of taxa >0.01% at the phylum level are showed by bar plot. **(B–E)** The taxonomic differences in colonic microbiota at the phylum levels. **(F)** The top 50 abundant taxa are shown by heatmap. **(G,H)** The taxomonic differences in colonic microbiota at the genus levels. CI7, CI21, and CI28 represent colonic samples obtained from IUGR piglets at 7, 21, and 28 days of age, respectively. CN7, CN21, and CN28 represent colonic samples obtained from NBW piglets at 7, 21, and 28 days of age, respectively. Red and blue represent the IUGR and NBW piglets, respectively. The data are expressed as means ± SEM (*n* = 8). ^*^p < 0.05; ^**^p < 0.01.

The colonic microbial compositions were analyzed at the phylum and genus levels (Figure 3). At the phylum level, in the IUGR and NBW piglets, Bacteroidetes (57.50% vs. 48.72%), Firmicutes (35.23% vs. 43.87%), and Proteobacteria (6.59% vs. 4.31%) were the top three phyla at 7 days of age; Bacteroidetes (43.64% vs. 58.75%), Firmicutes (45.94% vs. 35.15%), and Spirochaetae (4.94% vs. 2.36%) were the top three phyla at 21 days of age; and Bacteroidetes (29.99% vs. 38.67%), Firmicutes (60.60% vs. 51.57%), and Proteobacteria (3.63% vs. 3.54%) were the top three phyla at 28 days of age, respectively (Figure 3A). The relative abundance of Bacteroidetes was higher (*p* < 0.05), and Firmicutes and Synergistetes were lower (*p* < 0.05) in the IUGR piglets at 7 days of age compared with the NBW piglets (Figures 3B–D). However, the relative abundance of Bacteroidetes was lower (*p* < 0.05) in the IUGR piglets compared with the NBW piglets at 21 days of age (Figure 3E). However, there was no significant (*p* > 0.05) difference in the colonic microbial composition at the phylum level between the IUGR and NBW piglets at 28 days of age.

The top 50 most abundant genera in the colon of the IUGR and NBW piglets and their differences are presented in Figure 3F. At 7 days of age, *norank*_*S24*-*7* (18.79%) was the most abundant genus, followed by *Prevotella*_2 (9.90%), *Lactobacillus* (9.54%), *Bacteroides* (6.80%), and *Alloprevotella* (6.24%) in the IUGR piglets, whereas *norank_S24-7* (13.83%) was the most abundant genus, followed by *Rikenellaceae*_*RC9_gut_group* (9.38%), *Lactobacillus* (8.00%), *Bacteroides* (5.53%), and *Ruminococcaceae*_*UCG-002* (4.59%) in the NBW piglets. At 21 days of age, *Bacteroides* (9.29%) was the most abundant genus, followed by *norank_S24-7* (7.86%), *Lactobacillus* (7.86%), *norank_Erysipelotrichaceae* (5.36%), and *Prevotellaceae_NK3B31_group* (4.93%) in the IUGR piglets, whereas *norank_S24-7* (11.37%) was the most abundant genus, followed by *Bacteroides* (10.80%), *Prevotella*_2 (9.46%), *Alloprevotella* (7.37%), and *Prevotellaceae*_*NK3B31_group* (5.19%) in the NBW piglets. At 28 days of age, *[Eubacterium]*_*coprostanoligenes_group* (7.78%) was the most abundant genus, followed by *Lactobacillus* (6.87%), *Bacteroides* (6.00%), *norank_S24-7* (5.41%), and *Ruminococcaceae*_*NK4A214_group* (4.03%) in the IUGR piglets, while *norank_Bacteroides* (9.94%) was the most abundant genus, followed by *norank_S24-7* (5.95%), *norank_Erysipelotrichaceae* (4.07%), *norank*_*Lachnospiraceae* (3.87%), and *Prevotella*_2 (3.24%) in the NBW piglets (Figure 3F). The relative abundances of *Rikenellaceae_RC9_gut_group*, *Ruminococcaceae_UCG-002*, *Parabacteroides*, *norank_Bacteroidales*, *Cloacibacillus*, *Subdoligranulum*, *Howardella*, *unclassified_Peptostreptococcaceae*, *Pasteurella*, *Ruminococcaceae_UCG-003*, and *norank_Bradymonadales* were lower (*p* < 0.05), whereas *Enterococcus* was higher (*p* < 0.05) in the IUGR piglets at 7 days of age, when compared with the NBW piglets (Figure 3G). At 21 days of age, the relative abundances of *Phascolarctobacterium*, *Ruminococcaceae_UCG-005*, and *norank*_*Bradymonadales* were lower (*p* < 0.05), while *Roseburia* was higher (*p* < 0.05) in the IUGR piglets compared with the NBW piglets (Figure 3H). There was no significant (*p* > 0.05) difference in the colonic microbial composition between the IUGR and NBW piglets at 28 days of age.

### Differences in colonic microbial function between IUGR and NBW piglets

The PICRUSt1 was used to evaluate the metabolic function differences of gut microbiota in the colon of the IUGR and NBW piglets (Supplementary Figure 2). Compared with the NBW piglets, six pathways were up-regulated (*p* < 0.05) in the IUGR piglets at 7 days of age, including protein digestion and absorption, arachidonic acid metabolism, *Vibrio cholerae* pathogenic cycle, cellular antigens, toluene degradation, and ubiquinone/other terpenoid-quinone biosynthesis; seven pathways were down-regulated (*p* < 0.05), including arginine/proline metabolism, valine/leucine/isoleucine biosynthesis, pentose/glucuronate interconversions, chaperones and folding catalysts, butirosin and neomycin biosynthesis, phosphonate and phosphinate metabolism, and nicotinate and nicotinamide metabolism (Supplementary Figure 2A). At 21 days of age, 14 pathways were up-regulated (*p* < 0.05) in the IUGR piglets, including citrate cycle, tropane/piperidine/pyridine alkaloid biosynthesis, one carbon pool by folate, carbon fixation pathways in prokaryotes, RNA degradation, pantothenate and CoA biosynthesis, energy metabolism, glycine/serine/threonine metabolism, cysteine/methionine metabolism, translation proteins, galactose metabolism, starch and sucrose metabolism, pentose phosphate pathway, and transporters; six pathways were down-regulated (*p* < 0.05), including nitrotoluene degradation, arachidonic acid metabolism, isoquinoline alkaloid biosynthesis, biotin metabolism, riboflavin metabolism, and MAPK signaling pathway-yeast (Supplementary Figure 2B). However, there were no significant (*p* > 0.05) differences in the colonic functional metabolic pathways between the IUGR and NBW piglets at 28 days of age.

### Differences in colonic metabolome profiles between IUGR and NBW piglets

**Figure 4:**
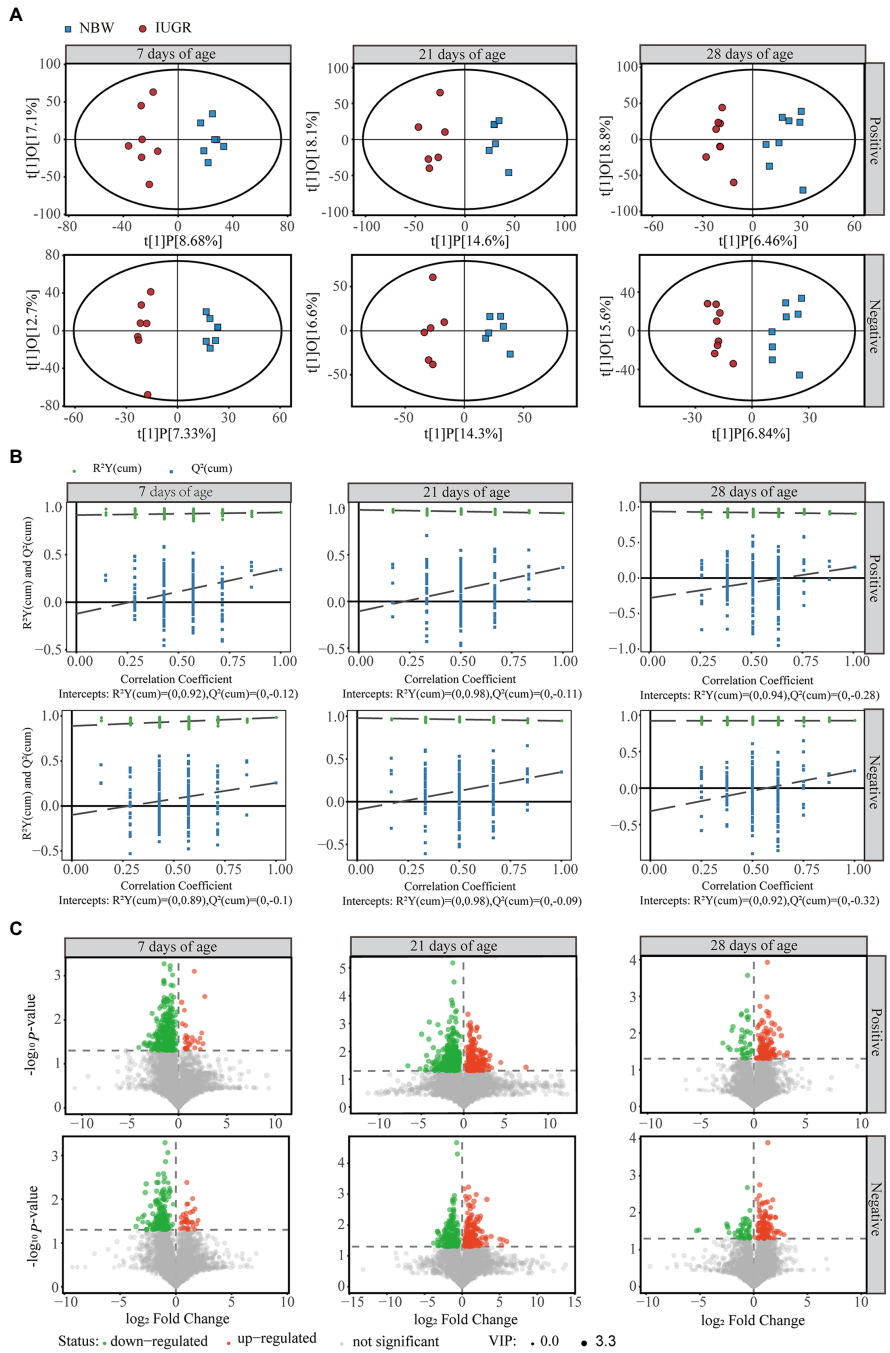
Differences in colonic metabolome profiles between the intrauterine growth restriction (IUGR) and normal birth weight (NBW) piglets at 7 (*n* = 7), 21 (*n* = 6), and 28 (*n* = 8) days of age. **(A)** The score plots of Orthogonal projections to latent structures-discriminant analysis (OPLS-DA) model based on the non-target metabolomics for the data from positive and negative ion modes of the colonic contents. Red and blue represent the IUGR and NBW piglets, respectively. **(B)** The permutation test results of OPLS-DA. **(C)** Volcano plots of positive and negative ion modes based on the non-target metabolomics of the colonic contents. Each symbol represents the identified metabolite, orange represents significantly up-regulation of different metabolites, green represents significantly down-regulation of different metabolites, and gray represents the metabolites those did not differ.

From the LC–MS/MS analysis, 8,322 and 6,132 valid peaks were obtained from the positive and negative ion modes, respectively. The unsupervised PCA was used to detect the overall changes in metabolic physiology of the IUGR and NBW piglets in the positive and negative ion modes (Supplementary Figure 3). The R^2^X value of the PCA model at 7, 21, and 28 days of age accounting for the variance was 0.580, 0.506, and 0.570 in the positive ion mode and 0.503, 0.528, and 0.555 in the negative ion mode, respectively (Supplementary Table 2). To maximize the discrimination between the IUGR and NBW piglets at 7, 21, and 28 days of age, the OPLS-DA was used to elucidate the different metabolic patterns (Figures 4A,B). The OPLS-DA models show goodness-of-fit (R^2^X and R^2^Y) and predictability (*Q*^2^), with 0.258, 0.994, and 0.344 in the positive ion mode and 0.200, 0.928, and 0.257 in the negative ion mode at 7 days of age; 0.327, 0.946, and 0.364 in the positive ion mode and 0.309, 0.947, and 0.347 in the negative ion mode at 21 days of age; and 0.253, 0.905, and 0.153 in the positive ion mode and 0.224, 0.925, and 0.239 in the negative ion mode at 28 days of age (Figure 4A; Supplementary Table 2), respectively. After 200 permutation tests, the *R*^2^ and *Q*^2^ intercept values at 7, 21, and 28 days of age were 0.92 and −0.12, 0.98 and −0.11, and 0.94 and −0.28 in the positive ion mode, respectively; whereas 0.89 and −0.10, 0.98 and −0.09, and 0.92 and −0.32 in the negative ion mode, respectively (Figure 4B). All samples were in the 95% confidence interval (Hotelling’s *t*-squared ellipse).

**Figure 5:**
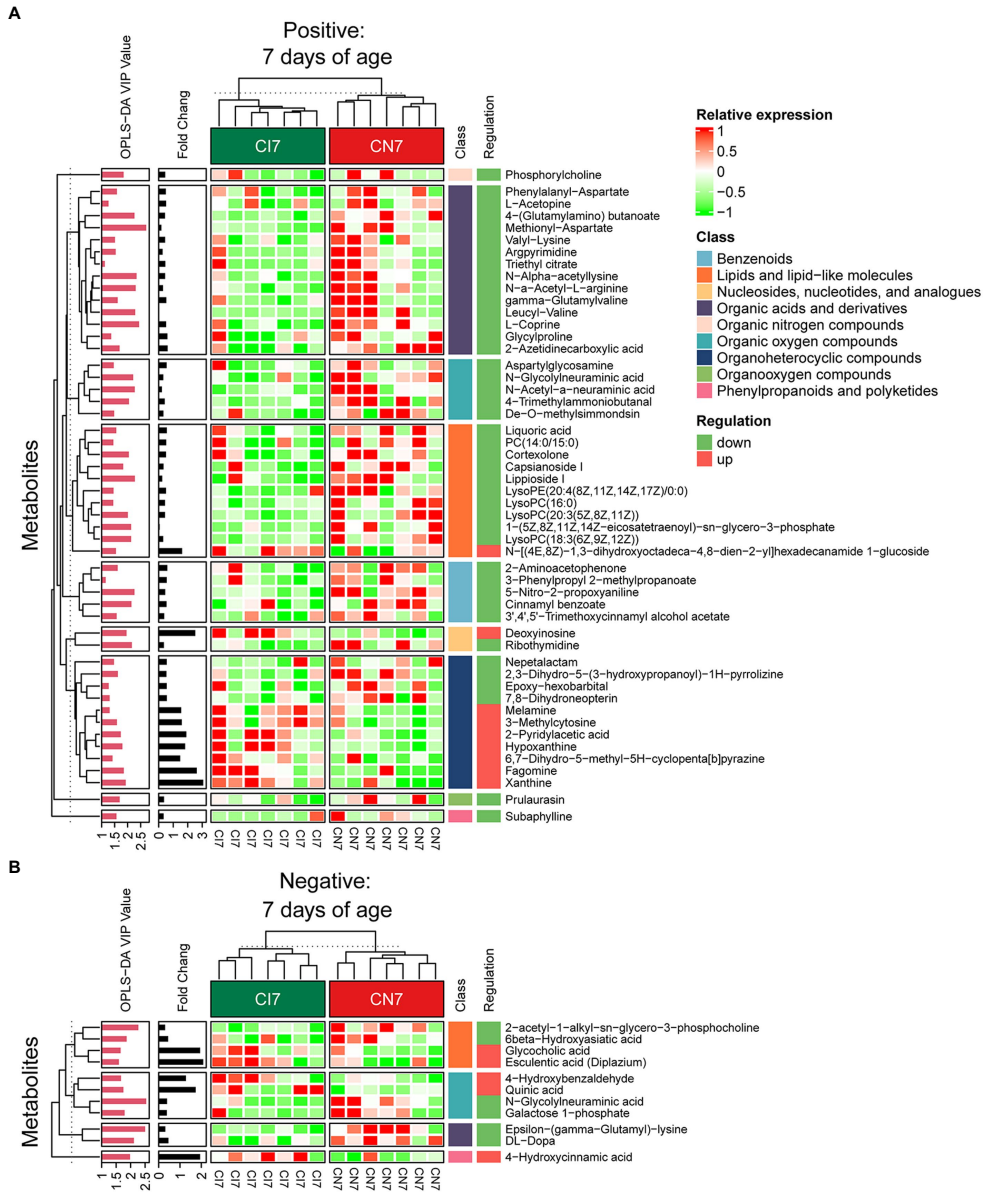
Hierarchical clustering analysis for different metabolites with MS2 based on the non-target metabolomics of the colonic contents between the intrauterine growth restriction (IUGR) and normal birth weight (NBW) piglets at 7 days of age (*n* = 7). **(A,B)** Represent positive and negative ion models, respectively. The relative metabolite level is depicted according to the color scale. Red indicates significant up-regulation (*p* < 0.05), and green indicates significant down-regulation (*p* < 0.05).

**Figure 6:**
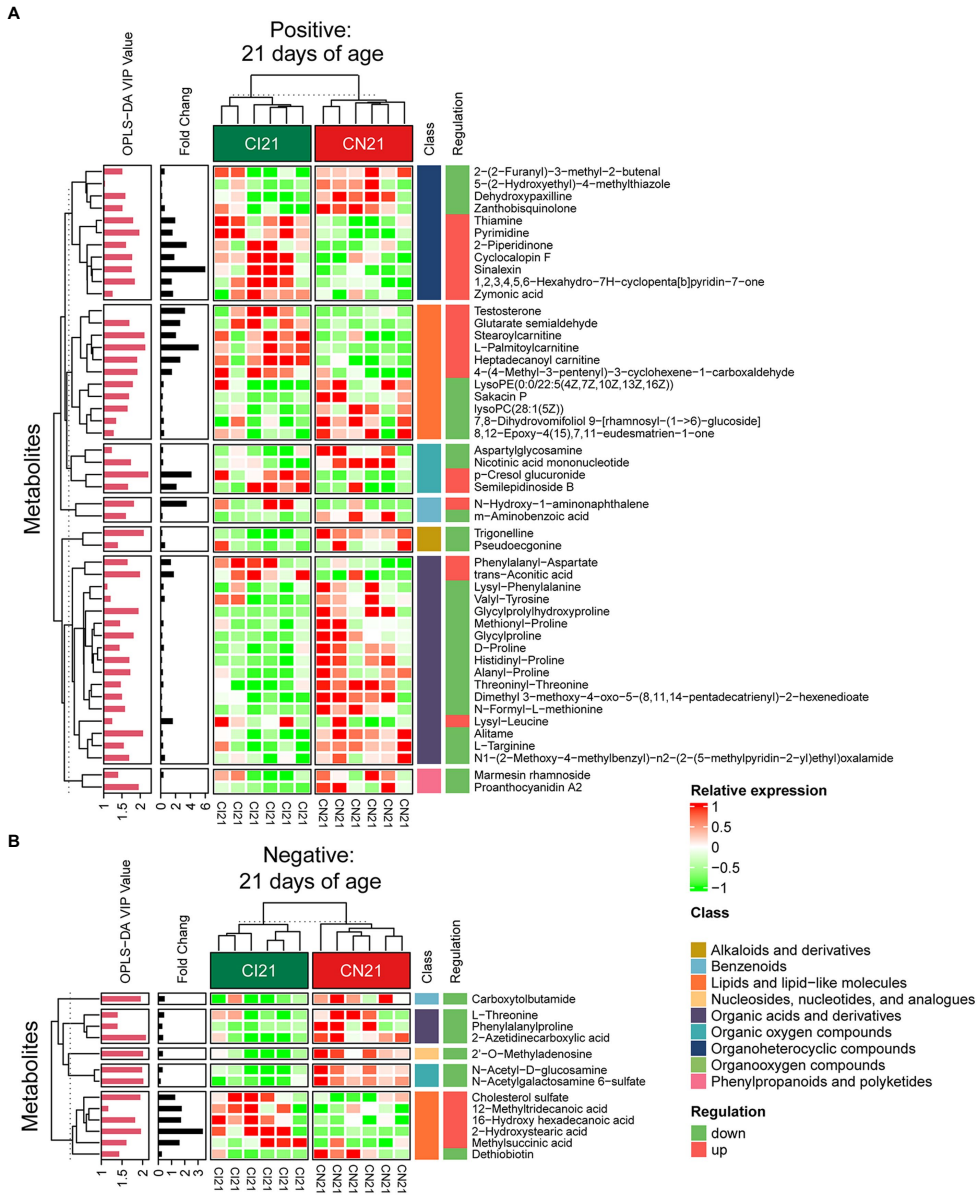
Hierarchical clustering analysis for different metabolites with MS2 based on the non-target metabolomics of the colonic contents between the intrauterine growth restriction (IUGR) and normal birth weight (NBW) piglets at 21 days of age (*n* = 6). **(A,B)** represent positive and negative ion models, respectively. The relative metabolite level is depicted according to the color scale. Red indicates significant up-regulation (*p* < 0.05), and green indicates significant down-regulation (*p* < 0.05).

**Figure 7:**
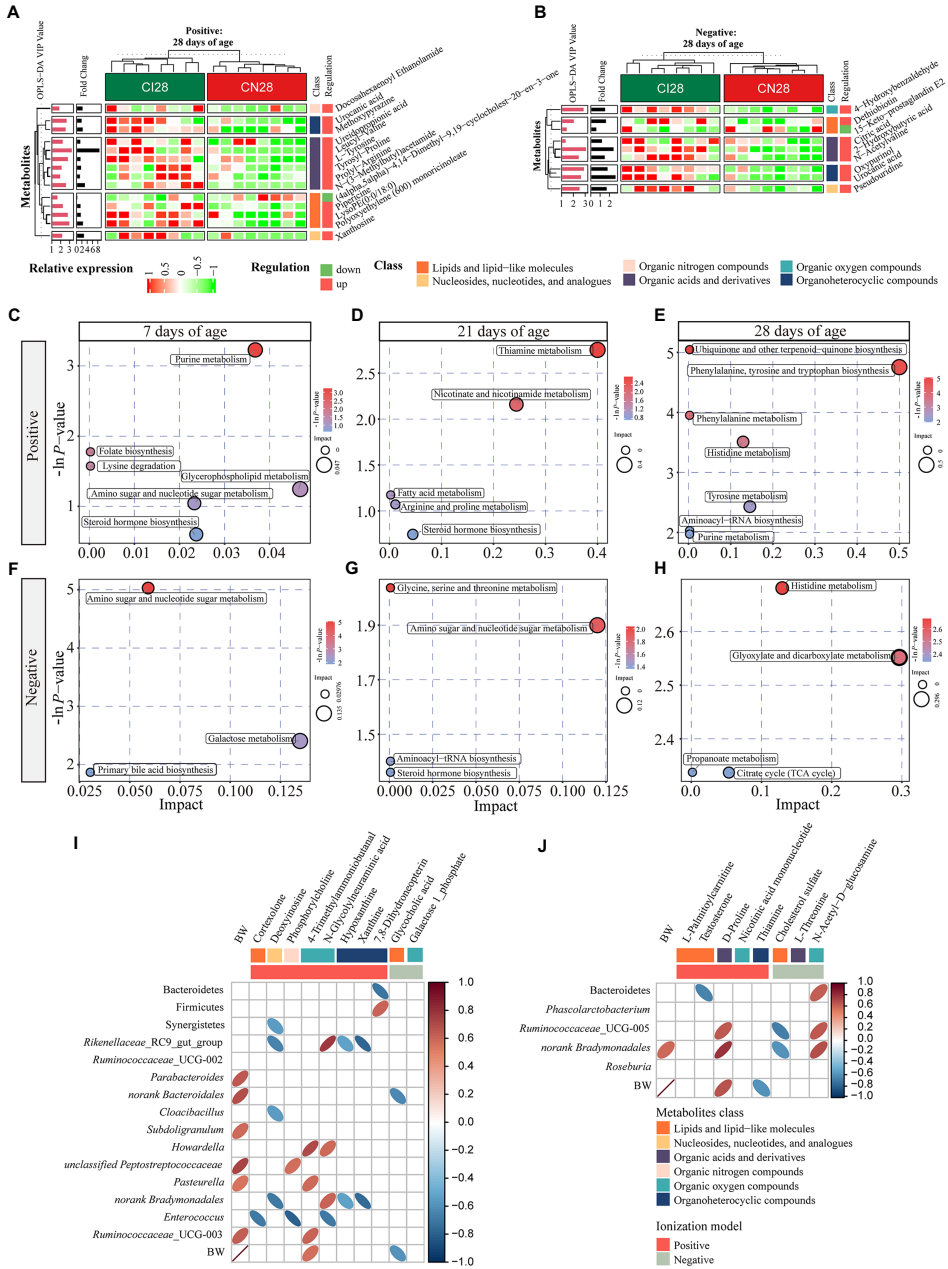
**(A,B)** Hierarchical clustering analysis for different metabolites with MS2 based on the non-target metabolomics of the colonic contents between the intrauterine growth restriction (IUGR) and normal birth weight (NBW) piglets at 28 days of age (*n* = 8; Positive: **A**, Negative: **B**). The relative metabolite level is depicted according to the color scale. Red indicates significant up-regulation (*p* < 0.05), and green indicates significant down-regulation (*p* < 0.05). **(C–H)** Metabolome view map of significant metabolic pathways characterized in the colonic contents between the IUGR and NBW piglets at 7, 21, and 28 days of age (Positive: **(C–E)**, Negative: **(F–H)**). Significantly changed pathways based on enrichment and topology analysis are shown. The x-axis represents pathway impact, and the y-axis represents pathway enrichment. Large size and red colors represent major pathway enrichment and high pathway impact values, respectively. **(I,J)** Spearman’s rank correlations between colonic microbiota and metabolites at 7 **(I)** and 21 **(J)** days of age. Ellipses in the graph represent significant correlations, red represents significant positive (*p* < 0.05) correlations, and blue represents significant negative (*p* < 0.05) correlations.

A total of 378, 611, and 191 differential metabolites in the positive ion mode and 195, 373, and 154 differential metabolites in the negative ion mode were quantified at 7, 21, and 28 days of age, respectively (VIP > 1 and *p* < 0.05; Figure 4C; Supplementary Table 3). After qualitative matching by MS2, 147 metabolites were identified as SDMs in the colon of the IUGR and NBW piglets (Figures 5, 6, 7A,B). These SDMs in the colonic contents belonged to alkaloids/derivatives, benzenoids, lipids/lipid-like molecules, nucleosides/nucleotides/analogs, organic acids/derivatives, organic nitrogen compounds, organic oxygen compounds, organoheterocyclic compounds, organooxygen compounds, and phenylpropanoids/polyketides were highlighted between the IUGR and NBW piglets. Compared with the NBW piglets, at 7 days of age, 9 and 5 SDMs were up-regulated (*p* < 0.05) in the IUGR piglets, whereas 42 and 6 SDMs were down-regulated (*p* < 0.05) in the positive (Figure 5A) and negative (Figure 5B) ion modes, respectively. At 21 days of age, 19 and 5 SDMs were up-regulated (*p* < 0.05) in the IUGR piglets, whereas 30 and 8 SDMs were down-regulated (*p* < 0.05) in the positive (Figure 6A) and negative (Figure 6B) ion modes, respectively, when compared with the NBW piglets. At 28 days of age, compared with the NBW piglets, 13 and 8 SDMs were up-regulated (*p* < 0.05) in the IUGR piglets, whereas 1 and 1 SDMs were down-regulated (*p* < 0.05) in the positive (Figure 7A) and negative (Figure 7B) ion modes, respectively.

The KEGG database was used to explore the metabolism pathways and metabolite markers in the IUGR piglets compared with the NBW piglets. A total of 22 metabolism pathways were enriched at 7, 21, and 28 days of age in the IUGR piglets compared with the NBW piglets (Figures 7C–H; Table 1). Six of these metabolic pathways were enriched (*p* < 0.05; Table 1). Compared with the NBW piglets, purine metabolism was up-regulated (*p* < 0.05) in the positive ion mode, whereas amino sugar and nucleotide sugar metabolism were down-regulated (*p* < 0.05) in the negative ion mode in the IUGR piglets at 7 days of age. At 21 days of age, there were no significant different metabolism pathways (*p* > 0.05). At 28 days of age, ubiquinone/other terpenoid-quinone biosynthesis, phenylalanine/tyrosine/tryptophan biosynthesis, phenylalanine metabolism, and histidine metabolism were up-regulated (*p* < 0.05) in the IUGR piglets in the positive ion mode compared with the NBW piglets (Figures 7C–H). These metabolism pathways included seven SDMs: xanthine, deoxyinosine, hypoxanthine, L-tyrosine, urocanic acid, galactose 1-phosphate, and N-glycolylneuraminic acid (Table 1).

**Table 1:** Metabolite pathways and significantly different metabolites (SDMs) markers between the IUGR and NBW piglets in the positive and negative ion models at 7, 21, and 28 days of age.

Ion model	Pathway	*p*-value	Impact	SDMs
7 days of age				
Positive	Purine metabolism	0.040	0.037	Xanthine; Deoxyinosine; Hypoxanthine
Positive	Folate biosynthesis	0.170	0.000	7,8-Dihydroneopterin
Positive	Lysine degradation	0.208	0.000	4-Trimethylammoniobutanal cpd
Positive	Glycerophospholipid metabolism	0.287	0.047	Phosphorylcholine
Positive	Amino sugar and nucleotide sugar metabolism	0.352	0.023	N-Glycolylneuraminic acid
Positive	Steroid hormone biosynthesis	0.548	0.024	Cortexolone
Negative	Amino sugar and nucleotide sugar metabolism	0.007	0.059	Galactose 1-phosphate; N-Glycolylneuraminic acid
Negative	Galactose metabolism	0.090	0.135	Galactose 1-phosphate
Negative	Primary bile acid biosynthesis	0.155	0.030	Glycocholic acid
21 days of age				
Positive	Thiamine metabolism	0.064	0.400	Thiamine
Positive	Nicotinate and nicotinamide metabolism	0.115	0.244	Nicotinic acid mononucleotide
Positive	Fatty acid metabolism	0.310	0.000	L-Palmitoylcarnitine
Positive	Arginine and proline metabolism	0.343	0.011	D-Proline
Positive	Steroid hormone biosynthesis	0.475	0.045	Testosterone
Negative	Glycine, serine, and threonine metabolism	0.131	0.000	L-Threonine
Negative	Amino sugar and nucleotide sugar metabolism	0.150	0.120	N-Acetyl-D-glucosamine
Negative	Aminoacyl-tRNA biosynthesis	0.247	0.000	L-Threonine
Negative	Steroid hormone biosynthesis	0.257	0.000	Cholesterol sulfate
28 days of age				
Positive	Ubiquinone and other terpenoid-quinone biosynthesis	0.006	0.000	L-Tyrosine
Positive	Phenylalanine, tyrosine and tryptophan biosynthesis	0.009	0.500	L-Tyrosine
Positive	Phenylalanine metabolism	0.019	0.000	L-Tyrosine
Positive	Histidine metabolism	0.030	0.130	Urocanic acid
Positive	Tyrosine metabolism	0.088	0.145	L-Tyrosine
Positive	Aminoacyl-tRNA biosynthesis	0.132	0.000	L-Tyrosine
Positive	Purine metabolism	0.140	0.000	Xanthosine
Negative	Histidine metabolism	0.069	0.130	Urocanic acid
Negative	Glyoxylate and dicarboxylate metabolism	0.078	0.296	Citric acid
Negative	Propanoate metabolism	0.097	0.000	2-Hydroxybutyric acid
Negative	Citrate cycle	0.097	0.054	Citric acid

IUGR, intrauterine growth restriction; NBW, normal birth weight.

### Correlation analysis among different microbiota, SDMs, and BW

Spearman’s correlations among the colonic microbiota abundance, SDMs, and BW (previously published from our research group; Zhang et al., 2019) are shown in Figures 7I,J. At 7 days of age (Figure 7I), the BW was positively correlated with *Parabacteroides*, *norank*_*Bacteroidales*, *Subdoligranulum*, *unclassified*_*Peptostreptococcaceae*, *Pasteurella*, and *Ruminococcaceae*_*UCG*-003 abundances and 4-trimethylammoniobutanal, while negatively correlated with glycocholic acid (*p* < 0.05). The cortexolone was negatively correlated with *Enterococcus* abundance (*p* < 0.05). Deoxyinosine was negatively correlated with Synergistetes, *Rikenellaceae*_*RC9_gut_group*, *Cloacibacillus,* and *norank*_*Bradymonadales* abundances (*p* < 0.05). The phosphorylcholine was positively correlated with *unclassified_Peptostreptococcaceae* but negatively correlated with *Enterococcus* abundances (*p* < 0.05), whereas 4-trimethylammoniobutanal was positively correlated with *Howardella*, *Pasteurella,* and *Ruminococcaceae*_*UCG*_003 abundances (*p* < 0.05). The N-glycolylneuraminic acid was positively correlated with *Rikenellaceae*_*RC9_gut_group*, *Howardella,* and *norank*_*Bradymonadales* abundances but negatively correlated with *Enterococcus* abundance (*p* < 0.05). Hypoxanthine and xanthine were negatively correlated with *Rikenellaceae*_*RC9*_*gut_group* and *norank*_*Bacteroidales* abundances (*p* < 0.05). Moreover, 7,8-dihydroneopterin was positively correlated with Bacteroidetes but negatively correlated with Firmicutes abundances (*p* < 0.05), whereas galactose 1-phosphate was negatively correlated with *norank*_*Bradymonadales* abundance (*p* < 0.05). At 21 days of age (Figure 7J), the BW was positively correlated with *norank*_*Bradymonadales* abundance and D-proline while negatively correlated with thiamine (*p* < 0.05). The testosterone was negatively correlated with Bacteroidetes abundance (*p* < 0.05), whereas D-proline was positively correlated with *Ruminococcaceae*_*UCG*-005 and *norank*_*Bradymonadales* abundances (*p* < 0.05). Moreover, cholesterol sulfate was negatively correlated with *Ruminococcaceae*_*UCG*-005 and *norank*_*Bradymonadales* abundances (*p* < 0.05), whereas N-acetyl-D-glutamate was positively correlated with *Bacteroidetes*, *Ruminococcaceae*_*UCG*-005, and *norank*_*Bradymonadales* abundances (*p* < 0.05).

### Differences in colonic short-chain fatty acids levels between IUGR and NBW piglets

Colonic SCFAs levels of the IUGR and NBW piglets at 7, 21, and 28 days of age are presented in Table 2. The levels of colonic isobutyrate at 7 days of age and isovalerate and total SCFAs at 21 days of age were lower (*p* < 0.05) in the IUGR piglets compared with the NBW piglets. In addition, the levels of acetate, propionate, butyrate, and total SCFAs were higher, whereas the level of isovalerate was lower in the IUGR piglets compared with the NBW piglets at 28 days of age (*p* < 0.05).

**Table 2:** Differences in colonic short-chain fatty acids (SCFAs) levels between the IUGR and NBW piglets (mg/g).

Items	7 days of age		21 days of age		28 days of age	
	NBW	IUGR	NBW	IUGR	NBW	IUGR
Acetate	1.10 ± 0.10	0.98 ± 0.07	1.33 ± 0.14	1.01 ± 0.10	2.19 ± 0.18	1.13 ± 0.12^**^
Propionate	0.42 ± 0.05	0.31 ± 0.04	0.48 ± 0.05	0.39 ± 0.05	0.76 ± 0.08	0.52 ± 0.05^*^
Isobutyrate	0.07 ± 0.01	0.04 ± 0.01^**^	0.07 ± 0.01	0.08 ± 0.01	0.06 ± 0.01	0.07 ± 0.01
Butyrate	0.16 ± 0.02	0.12 ± 0.02	0.18 ± 0.02	0.19 ± 0.02	0.29 ± 0.03	0.19 ± 0.03^*^
Isovalerate	0.09 ± 0.01	0.07 ± 0.01	0.16 ± 0.01	0.13 ± 0.01^*^	0.09 ± 0.01	0.13 ± 0.01^*^
Valerate	0.09 ± 0.01	0.07 ± 0.01	0.10 ± 0.01	0.10 ± 0.01	0.09 ± 0.01	0.09 ± 0.01
Total SCFAs	1.92 ± 0.16	1.58 ± 0.10	2.32 ± 0.16	1.90 ± 0.11^*^	3.48 ± 0.24	2.14 ± 0.12^**^

Data are presented as means ± SEM (*n* = 8). IUGR, intrauterine growth restriction; NBW, normal birth weight.

* *p* < 0.05;

** *p* < 0.01.

### Differences in the mRNA expression of colonic health-related genes between IUGR and NBW piglets

The mRNA expression of intestinal health-related genes of the IUGR and NBW piglets at 7, 21, and 28 days of age are shown in Table 3. Compared with the NBW piglets, colonic zonula occludens (*ZO*)*-1* expression in the IUGR piglets was down-regulated (*p* < 0.05) at 7 days of age. At 21 days of age, *ZO-1*, *occludin*, and interleukin (*IL*)*-4* expressions were down-regulated (*p* < 0.05) in the IUGR piglets compared with the NBW piglets. Moreover, tumor necrosis factor *(TNF)-α* and nuclear factor kappa B (*NF-κB*) expressions were up-regulated (*p* < 0.05) in the IUGR piglets compared with the NBW piglets at 28 days of age.

**Table 3:** Differences in mRNA expression of colonic mucosal genes related to barrier function between the IUGR and NBW piglets at 7, 21, and 28 days of age.

Items	7 days of age		21 days of age		28 days of age	
	NBW	IUGR	NBW	IUGR	NBW	IUGR
*ZO-1*	1.00 ± 0.06	0.81 ± 0.05^*^	1.00 ± 0.16	0.40 ± 0.10^**^	1.00 ± 0.14	0.67 ± 0.17
*Occludin*	1.00 ± 0.12	0.80 ± 0.07	1.00 ± 0.11	0.40 ± 0.07^***^	1.00 ± 0.19	0.78 ± 0.16
*IL-4*	1.00 ± 0.10	0.78 ± 0.10	1.00 ± 0.16	0.52 ± 0.08^*^	1.00 ± 0.24	0.47 ± 0.29
*TNF-α*	1.00 ± 0.11	0.78 ± 0.09	1.00 ± 0.14	1.07 ± 0.13	1.00 ± 0.07	1.41 ± 0.16^*^
*NF-κB*	1.00 ± 0.24	1.53 ± 0.22	1.00 ± 0.14	1.10 ± 0.20	1.00 ± 0.30	2.38 ± 0.12^**^

Data are presented as means ± SEM (*n* = 8). IUGR, intrauterine growth restriction; NBW, normal birth weight; ZO, zonula occludens; IL, interleukin; TNF-α, tumor necrosis factor alpha; NF-κB, nucelar factor kappa B.

* *p* < 0.05;

** *p* < 0.01;

*** *p* < 0.001.

## Discussion

The mammalian gut harbors a complex and diverse microbial community, which influences host’s normal physiology and disease susceptibility through their metabolic activity and the interactions with host (Rooks and Garrett, 2016). The present study compared the differences in colonic microbiota composition, metabolic profiles, and barrier function-related gene expression levels between the IUGR and NBW piglets. The findings indicate that the IUGR pigs present abnormal microbiota and nutrient metabolism in the colon, which may further affect the intestinal barrier function by regulating gene expression.

Gut microbial diversity is highly associated with the host’s health (Clarke et al., 2014). It has been reported that the higher diversity contributed to the gut microbiota maturation and host health (Le Chatelier et al., 2013). However, the decreased diversity of intestinal microbiota is considered a marker in gut dysbiosis (Clarke et al., 2014) and easily contributes to the increased risk of several intestine diseases (Weiss and Hennet, 2017). In the present study, the colonic microbial alpha diversity was decreased in the IUGR piglets at 7 and 21 days of age, which was consistent with our previous study on the small intestinal microbiota of IUGR piglets (Zhang et al., 2019), suggesting that the intestinal dysbiosis occurred in the IUGR piglets. In addition, the beta diversity analysis showed that colonic microbiota structure between the IUGR and NBW piglets were divided into two groups at 7, 21, and 28 days of age in the present study, indicating that the IUGR significantly altered the structure of colonic microbiota of piglets.

Bacteroidetes and Firmicutes were the two most predominant phyla in piglets (Li et al., 2018). In addition, Firmicutes/Bacteroidetes ratio plays an important role in digesting polysaccharide-rich diets and protecting against gut inflammation and colonic diseases (Ni et al., 2021). The sequencing of Bacteroidetes genomes confirms the presence of numerous carbohydrate-active enzymes for degrading high molecular weight organic matter, such as proteins and carbohydrates (Azad et al., 2018). Furthermore, Bacteroidetes play an important role in maintaining the host’s gut health by producing butyrate (Kim and Milner, 2007), which interact with the immune system to activate T-cell mediated responses (Wen et al., 2008) and prevent the potentially pathogenic bacteria from colonizing in the gut (Mazmanian et al., 2008). In the present study, a lower Bacteroidetes abundance was detected in the IUGR piglets at 21 days of age, suggesting the IUGR piglets had a lower efficiency of proteins and carbohydrates utilization from the diet compared with the NBW piglets. However, a higher Bacteroidetes proportion was found in the IUGR piglets at 7 days of age, which may be related to other bacteria composition changes in the IUGR piglets and still need further research. Firmicutes is associated with energy intake from diets (Turnbaugh et al., 2006), and a higher Firmicutes proportion was found in both obese children and adults (Ley et al., 2006). In the present study, a higher Firmicutes abundance was found in the NBW piglets at 7 days of age, suggesting that the IUGR piglets had lower efficiency of energy intake from the diets compared with the NBW piglets.

*Ruminococcaceae* contains numerous carbohydrate-active enzymes that can ferment the undigested complex carbohydrates and produce SCFAs, such as butyrate, which plays a key role in maintaining gut health (Biddle et al., 2013). In addition, a higher *Ruminococcaceae* abundance was detected in the obese mice (Kim et al., 2012). At the genus level, the present study found several low-abundant bacterial taxa, including *Ruminococcaceae_UCG-002*, *Ruminococcaceae_UCG-003*, and *Ruminococcaceae_UCG-005* in the colon of IUGR piglets at 7 and 21 days of age, implying that the IUGR did not allow the host to access indigestible energy sources to obtain extra energy for the growth and development. Some species of *Parabacteroides* genus, such as *P*. *goldsteinii* and *P*. *distasonis*, play a predominant role in anti-obesity effects (Wang et al., 2019; Wu et al., 2019). The present study showed that the *Parabacteroides* abundance in the IUGR piglets was higher compared to the NBW piglets, indicating that several members of *Parabacteroides* are negatively correlated with BW. In addition, *Subdoligranulum* and *Phascolarctobacterium* can produce SCFAs by fermenting carbohydrates to provide nutrients and energy for the host and play an important role in maintaining gut health (Deldot et al., 1993; Holmstrom et al., 2004). In the present study, colonic *Subdoligranulum* and *Phascolarctobacterium* had lower abundances in the IUGR piglets compared with the NBW piglets at 7 and 21 days of age, suggesting that the function of colonic epithelial cells of IUGR piglets may be adversely affected.

The PICRUSt1 is based on 16S rRNA gene sequencing to predicate the microbiota metabolic function (Langille et al., 2013). Amino acids are essential precursors for protein biosynthesis (Wu, 2009). Vitamins and cofactors are critical for converting nutrients to energy (Hu et al., 2016). In the present study, arginine/proline metabolism, valine/leucine/isoleucine biosynthesis, and nicotinate/nicotinamide metabolism were down-regulated in the IUGR piglets compared with the NBW piglets at 7 days of age, indicating that the growth and development might be suppressed in the IUGR piglets. Interestingly, amino acid, vitamin, glucose, and energy metabolisms were up-regulated in the IUGR piglets compared with the NBW piglets at 21 days of age; however, further in-depth studies are needed to explore the underlying mechanism of these metabolisms in IUGR piglets.

The colonic metabolome differences between the IUGR and NBW piglets were profiled using LC–MS/MS metabolomics analysis in the present study. According to HMDB database classification, the highest changing metabolites in the IUGR piglets compared with the NBW piglets were annotated to organic acids and their derivatives, which have been considered to be related to feed utilization in previous studies (Thacker et al., 1992). In animal production, organic acids are usually used as feed additives to promote growth, inhibit pathogens, and supply energy (Grilli et al., 2015). In addition, as important components of organic acids and their derivatives, amino acids can promote the intestinal development and health of piglets (Mou et al., 2019). Interestingly, in the present study, most of the SDMs of organic acids and derivatives in the colon of IUGR piglets were significantly decreased at 7 and 21 days of age while increased at 28 days of age compared with NBW piglets. Davila et al. (2013) reported that pigs with a high feed utilization rate had higher colonic concentrations of organic acids. These findings suggested that the feed utilization rate of IUGR piglets was lower, and this situation was alleviated with the increase of age.

In order to reveal the specific effect of SDMs in the colon of IUGR piglets, the KEGG database was used to characterize the most influential metabolism pathways. Recent studies showed that intestinal microbiota can improve experimental colitis in mice by regulating purine metabolism (Wu et al., 2020). In the present study, the purine metabolism was up-regulated in the IUGR piglets compared with the NBW piglets at 7 days of age, as well as the xanthine, hypoxanthine, and deoxyinosine metabolisms suggesting that inflammation might be present in the colon. The amino sugar/nucleotide sugar metabolism was negatively correlated with intestinal permeability (Liu et al., 2019). In the present study, the amino sugar/nucleotide sugar metabolism was down-regulated in the IUGR piglets compared with the NBW piglets at 7 days of age, as well as the galactose 1-phosphate and N-Glycolylneuraminic acid metabolisms, indicating that intestinal microbiota in the IUGR piglets were more likely to enter the circulation, resulting in dysregulated micro-ecological balance. However, there were no significantly different metabolic pathways in the IUGR piglets compared with the NBW piglets at 21 days of age. These findings were distinct from the predicted microbial functions analysis, suggesting the limitation and deviation of the analysis of the IUGR piglet’s colon microbial functions based solely on microbial data.

Ubiquinone is vital for cellular energy production (Søballe and Poole, 2000). Tyrosine is an essential amino acid for animals and can be replenished by the metabolic conversion of phenylalanine (Wang et al., 2022). In the present study, ubiquinone/other terpenoid-quinone biosynthesis, phenylalanine/tyrosine/tryptophan biosynthesis, phenylalanine metabolism, and histidine metabolism were up-regulated in the IUGR piglets compared with the NBW piglets at 28 days of age, as well as the L-tyrosine and urocanic acid indicating that the ability of the microbiota to utilize assimilable substrates is enhanced in IUGR piglets.

The SCFAs are produced by the microbial fermentation of undigested carbohydrates and nitrogenous substances (Rooks and Garrett, 2016). These metabolites are important for the host, such as inhibiting pathogenic bacteria growth, improving intestinal function, maintaining body fluid and electrolyte balances, and providing energy (Duncan et al., 2004; Tremaroli and Backhed, 2012). In the present study, the IUGR piglets had lower colonic isobutyrate level at 7 days of age, isovalerate and total SCFAs levels at 21 days of age, and acetate, propionate, butyrate, and total SCFAs levels at 28 days of age, suggesting that the colonic microbiota of IUGR piglets cannot effectively utilize carbohydrates and nitrogenous substances from diets. These findings were consistent with lower abundances of *Ruminococcaceae_UCG-002*, *Ruminococcaceae_UCG-003*, and *Ruminococcaceae_UCG-005* in the colon of IUGR piglets at 7 and 21 days of age in the present study. Moreover, the decreased SCFAs may also be related to the lower abundances of colonic Firmicutes and Bacteroidetes in the IUGR piglets, thus causing the IUGR piglets to be unable to obtain additional energy from these metabolites to meet their growth and development. However, the IUGR piglets had a higher isovalerate level at 28 days of age compared with the NBW piglets in the present study. The possible reason might be related to the age and gut maturation on energy absorption capacity from diets. However, it warranted further study to determine the exact mechanism.

The intestinal barrier can regulate the homeostasis of the body and resist the invasion of pathogens and foodborne antigens, which is important for the maintenance of the intestinal health of animals (Luissint et al., 2016). Tight junction proteins, such as *ZO*-1 and *occludin*, are key molecules that determine intestinal mucosal permeability (Zihni et al., 2016). In the present study, the IUGR piglets had lower mRNA expression levels of colonic *ZO-1* at 7 and 21 days of age and *occludin* at 21 days of age, suggesting that IUGR piglets had impaired intestinal integrity. This decrease may be related to the decrease in microbial diversity and the increase in pathogen abundance in the IUGR piglets (Zhang et al., 2019).

Cytokines are involved in the immune response and intestinal barrier function (Andrews et al., 2018). Inflammatory cytokines are closely related to the changes in tight junction proteins (Al-Sadi et al., 2009). In the present study, the IUGR piglets had a lower mRNA expression level of *IL-4* at 21 days of age while a higher *TNF-α* expression level at 28 days of age, suggesting that there was an intestinal inflammatory reaction in the IUGR piglets.

## Conclusion

In summary, the IUGR could affect intestinal micro-ecological dysbiosis of piglets during their early growth stage by decreasing the microbiota diversity and abundances, leading to impaired intestinal mucosal integrity. The IUGR was associated with the alterations of colonic microbial abundances of Bacteroidetes, Firmicutes, and other bacteria (taxonomically belong to the *Ruminococcaceae* family) that may be involved in the digestion, absorption, and metabolism of nutrients of the IUGR piglets. In addition, the IUGR piglets also showed dysregulated metabolomics profiles related to protein synthesis and the growth and development of piglets. Furthermore, there is a tight cross-talk between gut microbiota and metabolomic biomarkers. These findings will provide the crucial guiding significance for further research into dietary nutrients for IUGR piglets at the early stage.

## Data availability statement

The datasets presented in this study can be found in online repositories. The names of the repository/repositories and accession number(s) can be found at: NCBI Sequence Read Archive (SRA) with under the accession number PRJNA836103.

## Ethics statement

All aspects of this research were conducted in accordance with the Chinese Guidelines for Animal Welfare and was approved by the Animal Care and Use Committee of the Institute of Subtropical Agriculture, Chinese Academy of Sciences (No. ISA-2017-016).

## Author contributions

WT, WZ, and XK conceived and designed the experiment. WT, WZ, MA, CM, and QZ performed the experiment. WT and WZ processed the data. WT, WZ, and MA prepared and drafted the manuscript. MA and XK revised the manuscript. All authors contributed to the article and approved the submitted version.

## Funding

This study was jointly supported by the Special Funds for Construction of Innovative Provinces in Hunan Province (2019RS3022) and the National Natural Science Foundation of China (31772613).

## Conflict of interest

The authors declare that the research was conducted in the absence of any commercial or financial relationships that could be construed as a potential conflict of interest.

## Publisher’s note

All claims expressed in this article are solely those of the authors and do not necessarily represent those of their affiliated organizations, or those of the publisher, the editors and the reviewers. Any product that may be evaluated in this article, or claim that may be made by its manufacturer, is not guaranteed or endorsed by the publisher.

## Footnotes

^1^ https://www.ncbi.nlm.nih.gov/sra/PRJNA836103/

^2^ https://cloud.majorbio.com/

^3^ http://www.metaboanalyst.ca/

^4^ https://www.r-project.org/

## References

[ref1] Al-Sadi R. Boivin M. Ma T. (2009). Mechanism of cytokine modulation of epithelial tight junction barrier. Front. Biosci. 14, 2765–2778. doi: 10.2741/3413, PMID: 19273235 PMC3724223

[ref2] Andrews C. McLean M. H. Durum S. K. (2018). Cytokine tuning of intestinal epithelial function. Front. Immunol. 9:1270. doi: 10.3389/fimmu.2018.01270, PMID: 29922293 PMC5996247

[ref3] Azad M. A. K. Bin P. Liu G. Fang J. Li T. Yin Y. (2018). Effects of different methionine levels on offspring piglets during late gestation and lactation. Food Funct. 9, 5843–5854. doi: 10.1039/c8fo01343h, PMID: 30358792

[ref4] Bauer R. Walter B. Hoppe A. Gaser E. Lampe V. Kauf E.. (1998). Body weight distribution and organ size in newborn swine (*sus scrofa domestica*) – a study describing an animal model for asymmetrical intrauterine growth retardation. Exp. Toxicol. Pathol. 50, 59–65. doi: 10.1016/s0940-2993(98)80071-7, PMID: 9570503

[ref5] Biddle A. Stewart L. Blanchard J. Leschine S. (2013). Untangling the genetic basis of fibrolytic specialization by *Lachnospiraceae* and *Ruminococcaceae* in diverse gut communities. Diversity 5, 627–640. doi: 10.3390/d5030627

[ref6] Chassaing B. Van de Wiele T. De Bodt J. Marzorati M. Gewirtz A. T. (2017). Dietary emulsifiers directly alter human microbiota composition and gene expression *ex vivo* potentiating intestinal inflammation. Gut 66, 1414–1427. doi: 10.1136/gutjnl-2016-313099, PMID: 28325746 PMC5940336

[ref7] Clarke S. F. Murphy E. F. O'Sullivan O. Lucey A. J. Humphreys M. Hogan A.. (2014). Exercise and associated dietary extremes impact on gut microbial diversity. Gut 63, 1913–1920. doi: 10.1136/gutjnl-2013-306541, PMID: 25021423

[ref8] Davila A. M. Blachier F. Gotteland M. Andriamihaja M. Benetti P. H. Sanz Y.. (2013). Intestinal luminal nitrogen metabolism: role of the gut microbiota and consequences for the host. Pharmacol. Res. 68, 95–107. doi: 10.1016/j.phrs.2012.11.005, PMID: 23183532

[ref9] Deldot T. Osawa R. Stackebrandt E. (1993). *Phascolarctobacterium faecium* gen-nov, spec nov, a novel taxon of the *Sporomusa* group of bacteria. Sys. Appl. Microbiol. 16, 380–384. doi: 10.1016/S0723-2020(11)80269-9

[ref10] D'Inca R. Kloareg M. Gras-Le Guen C. Le Huerou-Luron I. (2010). Intrauterine growth restriction modifies the developmental pattern of intestinal structure, transcriptomic profile, and bacterial colonization in neonatal pigs. J. Nutr. 140, 925–931. doi: 10.3945/jn.109.116822, PMID: 20335628

[ref11] Dong L. Zhong X. He J. T. Zhang L. L. Bai K. W. Xu W.. (2016). Supplementation of tributyrin improves the growth and intestinal digestive and barrier functions in intrauterine growth-restricted piglets. Clin. Nutr. 35, 399–407. doi: 10.1016/j.clnu.2015.03.002, PMID: 26112894

[ref12] Duan Y. H. Zeng L. M. Li F. N. Wang W. L. Li Y. H. Guo Q. P.. (2017). Effect of branched-chain amino acid ratio on the proliferation, differentiation, and expression levels of key regulators involved in protein metabolism of myocytes. Nutrition 36, 8–16. doi: 10.1016/j.nut.2016.10.016, PMID: 28336113

[ref13] Duncan S. H. Holtrop G. Lobley G. E. Calder A. G. Stewart C. S. Flint H. J. (2004). Contribution of acetate to butyrate formation by human faecal bacteria. Br. J. Nutr. 91, 915–923. doi: 10.1079/bjn20041150, PMID: 15182395

[ref14] Edgar R. C. (2013). UPARSE: highly accurate OTU sequences from microbial amplicon reads. Nat. Methods 10, 996–998. doi: 10.1038/nmeth.2604, PMID: 23955772

[ref15] Edgar R. C. (2016). UNOISE2: improved error-correction for Illumina 16S and ITS amplicon sequencing. *BioRXiv*. 081257. doi: 10.1101/081257

[ref16] Ferenc K. Pilzys T. Skrzypek T. Garbicz D. Marcinkowski M. Dylewska M.. (2017). Structure and function of enterocyte in intrauterine growth retarded pig neonates. Dis. Markers 2017:5238134. doi: 10.1155/2017/5238134, PMID: 28757676 PMC5516756

[ref17] Gilbert W. M. Danielsen B. (2003). Pregnancy outcomes associated with intrauterine growth restriction. Am. J. Obstet. Gynecol. 188, 1596–1601. doi: 10.1067/mob.2003.384 12824998

[ref18] Grilli E. Tugnoli B. Passey J. L. Stahl C. H. Piva A. Moeser A. J. (2015). Impact of dietary organic acids and botanicals on intestinal integrity and inflammation in weaned pigs. BMC Vet. Res. 11:96. doi: 10.1186/s12917-015-0410-0, PMID: 25889654 PMC4483210

[ref19] Holmstrom K. Collins M. D. Moller T. Falsen E. Lawson P. A. (2004). *Subdoligranulum variable* gen. nov., sp nov from human feces. Anaerobe 10, 197–203. doi: 10.1016/j.anaerobe.2004.01.004, PMID: 16701519

[ref20] Hu J. Nie Y. Chen J. Zhang Y. Wang Z. Fan Q.. (2016). Gradual changes of gut microbiota in weaned miniature piglets. Front. Microbiol. 7:1727. doi: 10.3389/fmicb.2016.01727, PMID: 27853453 PMC5090779

[ref21] Jiang L. Feng C. Tao S. Li N. Zuo B. Han D.. (2019). Maternal imprinting of the neonatal microbiota colonization in intrauterine growth restricted piglets: a review. J. Anim. Sci. Biotechnol. 10:88. doi: 10.1186/s40104-019-0397-7, PMID: 31737268 PMC6844051

[ref22] Kamada N. Chen G. Y. Inohara N. Nunez G. (2013). Control of pathogens and pathobionts by the gut microbiota. Nat. Immunol. 14, 685–690. doi: 10.1038/ni.2608, PMID: 23778796 PMC4083503

[ref23] Kim K. A. Gu W. Lee I. A. Joh E. H. Kim D. H. (2012). High fat diet-induced gut microbiota exacerbates inflammation and obesity in mice via the TLR4 signaling pathway. PLoS One 7:e47713. doi: 10.1371/journal.pone.0047713, PMID: 23091640 PMC3473013

[ref24] Kim Y. S. Milner J. A. (2007). Dietary modulation of colon cancer risk. J. Nutr. 137, 2576S–2579S. doi: 10.1093/jn/137.11.2576S 17951506

[ref25] Langille M. G. I. Zaneveld J. Caporaso J. G. McDonald D. Knights D. Reyes J. A.. (2013). Predictive functional profiling of microbial communities using 16S rRNA marker gene sequences. Nat. Biotechnol. 31, 814–821. doi: 10.1038/nbt.2676, PMID: 23975157 PMC3819121

[ref26] Le Chatelier E. Nielsen T. Qin J. J. Prifti E. Hildebrand F. Falony G.. (2013). Richness of human gut microbiome correlates with metabolic markers. Nature 500, 541–546. doi: 10.1038/nature12506, PMID: 23985870

[ref27] Ley R. E. Turnbaugh P. J. Klein S. Gordon J. I. (2006). Microbial ecology – human gut microbes associated with obesity. Nature 444, 1022–1023. doi: 10.1038/4441022a 17183309

[ref28] Li N. Huang S. M. Jiang L. L. Wang W. Li T. T. Zuo B.. (2018). Differences in the gut microbiota establishment and metabolome characteristics between low- and normal-birth-weight piglets during early-life. Front. Microbiol. 9:1798. doi: 10.3389/fmicb.2018.01798, PMID: 30245669 PMC6137259

[ref29] Littman D. R. Pamer E. G. (2011). Role of the commensal microbiota in normal and pathogenic host immune responses. Cell Host Microbe 10, 311–323. doi: 10.1016/j.chom.2011.10.004, PMID: 22018232 PMC3202012

[ref30] Liu X. Zheng H. Lu R. Huang H. Zhu H. Yin C.. (2019). Intervening effects of total alkaloids of bunting on rats with antibiotic-induced gut microbiota dysbiosis based on 16S rRNA gene sequencing and untargeted metabolomics analyses. Front. Microbiol. 10:1151. doi: 10.3389/fmicb.2019.01151, PMID: 31214133 PMC6555270

[ref31] Luissint A. C. Parkos C. A. Nusrat A. (2016). Inflammation and the intestinal barrier: leukocyte-ypithelial nell interactions, cell junction remodeling, and mucosal repair. Gastroenterology 151, 616–632. doi: 10.1053/j.gastro.2016.07.008, PMID: 27436072 PMC5317033

[ref32] Magoc T. Salzberg S. L. (2011). FLASH: fast length adjustment of short reads to improve genome assemblies. Bioinformatics 27, 2957–2963. doi: 10.1093/bioinformatics/btr507, PMID: 21903629 PMC3198573

[ref33] Marchesi J. R. Adams D. H. Fava F. Hermes G. D. A. Hirschfield G. M. Hold G.. (2016). The gut microbiota and host health: a new clinical frontier. Gut 65, 330–339. doi: 10.1136/gutjnl-2015-309990, PMID: 26338727 PMC4752653

[ref34] Matamoros S. Gras-Leguen C. Le Vacon F. Potel G. de La Cochetiere M. F. (2013). Development of intestinal microbiota in infants and its impact on health. Trends Microbiol. 21, 167–173. doi: 10.1016/j.tim.2012.12.001 23332725

[ref35] Mazmanian S. K. Round J. L. Kasper D. L. (2008). A microbial symbiosis factor prevents intestinal inflammatory disease. Nature 453, 620–625. doi: 10.1038/nature07008, PMID: 18509436

[ref36] Mou Q. Yang H. S. Yin Y. L. Huang P. F. (2019). Amino acids influencing intestinal development and health of the piglets. Animals 9:302. doi: 10.3390/ani9060302, PMID: 31159180 PMC6617173

[ref37] Ni H. Long L. Bin P. Azad M. A. K. Xu K. Zhou X.. (2021). Maternal cysteine intake influenced oxidative status and lipid-related gut microbiota and plasma metabolomics in male suckling piglets. Anim. Feed Sci. Technol. 276:114947. doi: 10.1016/j.anifeedsci.2021.114947

[ref38] Nicholson J. K. Holmes E. Kinross J. Burcelin R. Gibson G. Jia W.. (2012). Host-gut microbiota metabolic interactions. Science 336, 1262–1267. doi: 10.1126/science.1223813 22674330

[ref39] Rooks M. G. Garrett W. S. (2016). Gut microbiota, metabolites and host immunity. Nat. Rev. Immunol. 16, 341–352. doi: 10.1038/nri.2016.42, PMID: 27231050 PMC5541232

[ref40] Schmittgen T. D. Livak K. J. (2008). Analyzing real-time PCR data by the comparative C_T_ method. Nat. Protoc. 3, 1101–1108. doi: 10.1038/nprot.2008.73, PMID: 18546601

[ref41] Sciascia Q. Das G. Metges C. C. (2016). Review: the pig as a model for humans: effects of nutritional factors on intestinal function and health. J. Anim. Sci. 94, 441–452. doi: 10.2527/jas.2015-9788

[ref42] Søballe B. Poole R. K. (2000). Ubiquinone limits oxidative stress in *Escherichia coli*. Microbiology 146, 787–796. doi: 10.1099/00221287-146-4-787, PMID: 10784036

[ref43] Sonnenburg J. L. Backhed F. (2016). Diet-microbiota interactions as moderators of human metabolism. Nature 535, 56–64. doi: 10.1038/nature18846, PMID: 27383980 PMC5991619

[ref44] Thacker P. A. Campbell G. L. Grootwassink J. (1992). The effect of organic-acids and enzyme supplementation on the performance of pigs fed barley-based diets. Can. J. Anim. Sci. 72, 395–402. doi: 10.4141/cjas92-047

[ref45] Tremaroli V. Backhed F. (2012). Functional interactions between the gut microbiota and host metabolism. Nature 489, 242–249. doi: 10.1038/nature11552 22972297

[ref46] Turnbaugh P. J. Ley R. E. Mahowald M. A. Magrini V. Mardis E. R. Gordon J. I. (2006). An obesity-associated gut microbiome with increased capacity for energy harvest. Nature 444, 1027–1031. doi: 10.1038/nature05414, PMID: 17183312

[ref47] Valdes A. M. Walter J. Segal E. Spector T. D. (2018). Role of the gut microbiota in nutrition and health. BMJ 361:k2179. doi: 10.1136/bmj.k2179, PMID: 29899036 PMC6000740

[ref48] Wang Q. Garrity G. M. Tiedje J. M. Cole J. R. (2007). Naive Bayesian classifier for rapid assignment of rRNA sequences into the new bacterial taxonomy. Appl. Environ. Microbiol. 73, 5261–5267. doi: 10.1128/aem.00062-07, PMID: 17586664 PMC1950982

[ref49] Wang K. Liao M. F. Zhou N. Bao L. Ma K. Zheng Z. Y.. (2019). Parabacteroides distasonis alleviates obesity and metabolic dysfunctions via production of succinate and secondary bile acids. Cell Rep. 26, 222.e5–235.e5. doi: 10.1016/j.celrep.2018.12.028, PMID: 30605678

[ref50] Wang C. Wei S. Jin M. Liu B. Yue M. Wang Y. (2022). Integrated microbiomic and metabolomic dynamics of fermented corn and soybean by-product mixed substrate. Front. Nutr. 9:831243. doi: 10.3389/fnut.2022.831243, PMID: 35299761 PMC8922052

[ref51] Weiss G. A. Hennet T. (2017). Mechanisms and consequences of intestinal dysbiosis. Cell. Mol. Life Sci. 74, 2959–2977. doi: 10.1007/s00018-017-2509-x 28352996 PMC11107543

[ref52] Wen L. Ley R. E. Volchkov P. Y. Stranges P. B. Avanesyan L. Stonebraker A. C.. (2008). Innate immunity and intestinal microbiota in the development of type 1 diabetes. Nature 455, 1109–1113. doi: 10.1038/nature07336, PMID: 18806780 PMC2574766

[ref53] Wu G. Y. (2009). Amino acids: metabolism, functions, and nutrition. Amino Acids 37, 1–17. doi: 10.1007/s00726-009-0269-0 19301095

[ref54] Wu G. Y. Bazer F. W. Cudd T. A. Meininger C. J. Spencer T. E. (2004). Maternal nutrition and fetal development. J. Nutr. 134, 2169–2172. doi: 10.1093/jn/134.9.2169 15333699

[ref55] Wu G. Bazer F. W. Wallace J. M. Spencer T. E. (2006). BOARD-INVITED REVIEW: intrauterine growth retardation: implications for the animal sciences. J. Anim. Sci. 84, 2316–2337. doi: 10.2527/jas.2006-156, PMID: 16908634

[ref56] Wu T. R. Lin C. S. Chang C. J. Lin T. L. Martel J. Ko Y. F.. (2019). Gut commensal *Parabacteroides goldsteinii* plays a predominant role in the anti-obesity effects of polysaccharides isolated from *Hirsutella sinensis*. Gut 68, 248–262. doi: 10.1136/gutjnl-2017-315458, PMID: 30007918

[ref57] Wu J. W. Wei Z. H. Cheng P. Qian C. Xu F. M. Yang Y.. (2020). Rhein modulates host purine metabolism in intestine through gut microbiota and ameliorates experimental colitis. Theranostics. 10, 10665–10679. doi: 10.7150/thno.43528, PMID: 32929373 PMC7482825

[ref58] Zhang W. Ma C. Xie P. Zhu Q. Wang X. Yin Y.. (2019). Gut microbiota of newborn piglets with intrauterine growth restriction have lower diversity and different taxonomic abundances. J. Appl. Microbiol. 127, 354–369. doi: 10.1111/jam.14304, PMID: 31077497 PMC6916403

[ref59] Zhou X. L. Kong X. F. Yang X. J. Yin Y. L. (2012). Soybean oligosaccharides alter colon short-chain fatty acid production and microbial population *in vitro*. J. Anim. Sci. 90, 37–39. doi: 10.2527/jas.50269 23365277

[ref60] Zihni C. Mills C. Matter K. Balda M. S. (2016). Tight junctions: from simple barriers to multifunctional molecular gates. Nat. Rev. Mol. Cell Biol. 17, 564–580. doi: 10.1038/nrm.2016.80, PMID: 27353478

