# Supplementary material for: Metabolome, microbiome, and gene expression alterations in the colon of newborn piglets with intrauterine growth restriction
Source: Front Microbiol. 2022 Sep 14;13:989060. doi: 10.3389/fmicb.2022.989060 (PMC9518826; doi:10.3389/fmicb.2022.989060)
Supplement: Supplementary file 1 [file Data_Sheet_1.PDF]

Supplementary Materials

Supplementary Table 1 Primer sequence used for real-time PCR analysis.

Items	Primer Sequence（5′-3′）	Accession ID	Product size (bp)
IL-4	F: GCTTCGGCACATCTACAGACACC<br>R: TCTTGGCTTCATGCACAGAACAGG	NM 214123.1	110
NF-κB	F: TGCCAGACACAGATGACCG<br>R: ATGGCGTAAAGGGATAGGGC	NM 001114281.1	195
Occludin	F: CAGTGGTAACTTGGAGGCGTCTTC<br>R: CGTGTAGTCTGTCTCGTAATGGTCTTG	XM 005672525.3	100
TNF-α	F: GCACTGAGAGCATGATCCGAGAC<br>R: CGACCAGGAGGAAGGAGAAGAGG	NM 214022.1	120
ZO-1	F: TCCTGAGTTTGATAGTGGCGTTGAC<br>R: CACGGTGTGACCATCCTCATCTTC	XM 021098896.1	148
β-Actin	F: GATCTGGCACCACACCTTCTACAAC<br>R: TCATCTTCTCACGGTTGGCTTTGG	XM 021086047.1	107

Supplementary Table 2 The PCA and OPLS-DA model parameters between the IUGR and NBW piglets.

Items	Type	R^2X	R^2Y	Q^2
Positive ion model				
CI7 vs CN7	PCA	0.580		
CI21 vs CN21	PCA	0.506		
CI28 vs CN28	PCA	0.570		
CI7 vs CN7	OPLS-DA	0.258	0.944	0.344
CI21 vs CN21	OPLS-DA	0.327	0.946	0.364
CI28 vs CN28	OPLS-DA	0.253	0.905	0.153
Negative ion model				
CI7 vs CN7	PCA	0.503		
CI21 vs CN21	PCA	0.528		
CI28 vs CN28	PCA	0.550		
CI7 vs CN7	OPLS-DA	0.200	0.982	0.257
CI21 vs CN21	OPLS-DA	0.309	0.947	0.347
CI28 vs CN28	OPLS-DA	0.224	0.925	0.239

PCA, principal component analysis; OPLS-DA, orthogonal projections to latent structures-discriminant analysis; IUGR, intrauterine growth restriction; NBW, normal birth weight.

Supplementary Table 3 Different numbers of metabolites in the colonic contents between the IUGR and NBW piglets.

Items	Ion model	Different metabolites	Up-regulated	Down-regulated
CI7 vs CN7	Positive	378	29	349
CI21 vs CN21	Positive	611	240	371
CI28 vs CN28	Positive	191	151	40
CI7 vs CN7	Negative	195	34	161
CI21 vs CN21	Negative	373	193	180
CI28 vs CN28	Negative	154	103	51

IUGR, intrauterine growth restriction; NBW, normal birth weight.

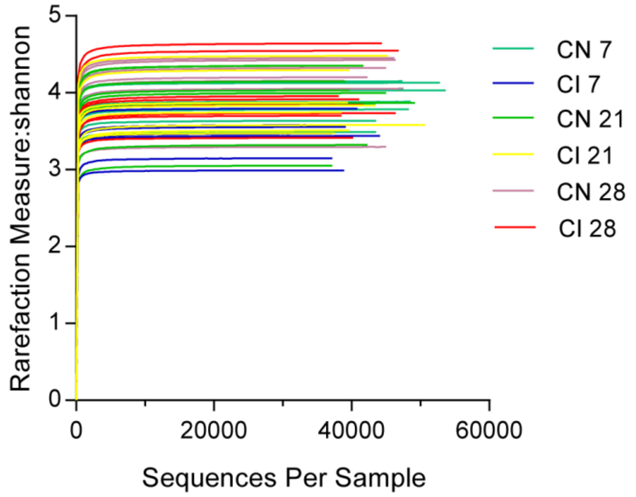

Supplementary Figure 1 The microbiota sequences rarefaction curve drawn with the Shannon index.

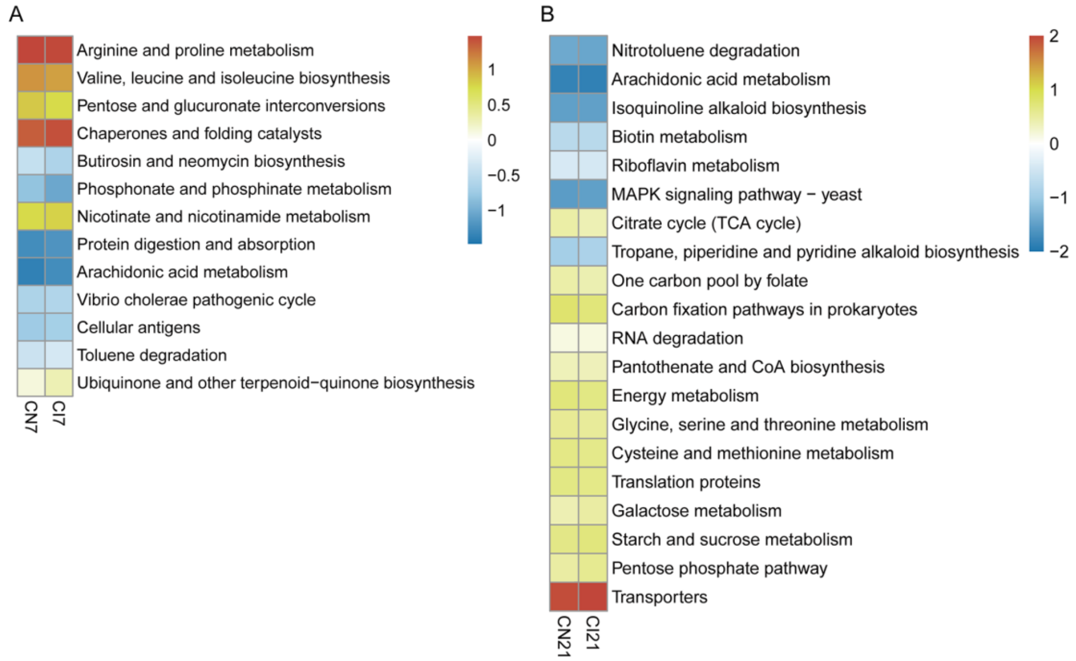

Supplementary Figure 2 Differences in function of gut microbiota between the intrauterine growth restriction (IUGR) and normal birth weight (NBW) piglets (*n* = 8). The average numbers of KEGG pathway (level 3) in each group were lg transformed and shown in the heatmap. (A) and (B): Differences in abundance of colon microbial pathways between the NBW and IUGR piglets at 7 and 21 days of age, respectively. CN7 and CN21 represent colonic samples obtained from NBW piglets at 7 and 21 days of age, respectively. CI7 and CI21 represent colonic samples obtained from IUGR piglets at 7 and 21 days of age, respectively.

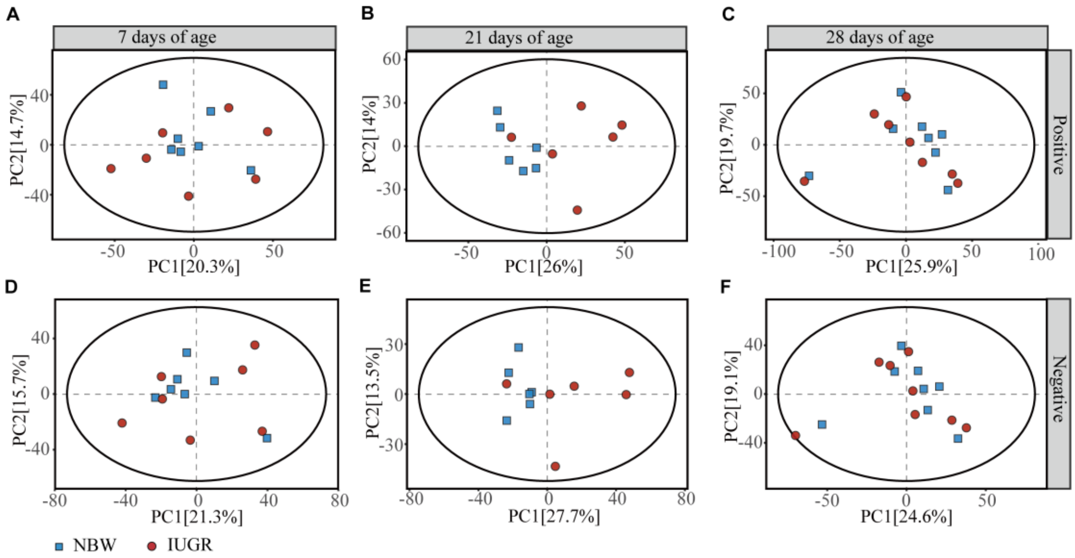

Supplementary Figure 3 Principal component analysis (PCA) model based on the non-target metabolomics for the data from positive (A–C) and negative (D–F) ion models for the colonic contents between the intrauterine growth restriction (IUGR) and normal birth weight (NBW) piglets at 7 (n = 7), 21 (n = 6), and 28 (n = 8) days of age. Red and blue represent the IUGR and NBW piglets, respectively.
